# The Hindu Lying-in Chamber

**Published:** 1898-04

**Authors:** 


### Selections and Abstracts.

### THE HINDU LYING-IN CHAMBER.

In the last meeting of the State Medical Society I took the position that the usually accepted opinion among physicians of a low mortality rate among child-bearing women of uncivilized people was an error. Their ignorant practices, their filthiness, their superstition, the woful absence of any except instinctive knowledge, lead us to expect a high mortality. Any other result would set at naught all our theories of asepsis. The testimony of competent observers establishes this position; this account of the Hindu Lying-in Chamber confirms it.

"The mortality in childbed among Hindu women is notoriously high, a circumstance which is no doubt largely due to the very early age at which they become mothers. A still more potent cause, however, is to be found in the shocking, and apparently deliberate barbarity with which they are treated during the puerperium. The lying-in chamber of a Hindu family is ordinarily a little, damp, ill-ventilated hut or room in some remote corner of the courtyard or compound. In this the expectant mother is placed, and there she remains from eleven to thirty-one days, during which, according to Brahminical law, she is looked upon as unclean. There is only one small inlet to this apartment and the door is carefully closed to exclude those evil spirits, light and air. In order probably to purify the unfortunate women by heat, wood fires are kept burning in the room both night and day. The smoke has to find its way to the outer air as best it can through any chinks there may happen to be in the roof or walls, which are usually made of bamboo with a thatching of mats or straw. With the view of more effectually exorcising the unclean spirits a powder composed of peppercorns or ginger is given to the patient during the first few days; this preparation is administered either in the form of a paste or dissolved in boiling water as a tisane. It is not surprising to learn that the result of this elaborately perverse therapeusis is that something like 40 per cent. of the women subjected to it die of puerperal fever and tetanus within the first fortnight after delivery."—*Pacific Medical Journal.*

